# Medicalization of female genital mutilation in Egypt: Trends, drivers, and prospects for elimination

**DOI:** 10.1371/journal.pgph.0005195

**Published:** 2025-11-20

**Authors:** Shatha Elnakib, May Sallab, Amira Hussein, Meral Marouf, Shadia Elshiwy, Reem Elsherbini, Kirantheja Daggula, Indira Prihartono

**Affiliations:** 1 Department of International Health, Johns Hopkins University Bloomberg School of Public Health, Baltimore, Maryland, United States of America; 2 UNFPA Egypt Country Office, Cairo, Egypt; 3 Independent Consultant, Cairo, Egypt; 4 UNFPA Arab States Regional Office, Cairo, Egypt; 5 UNICEF, Cairo, Egypt; UNAM: Universidad Nacional Autonoma de Mexico, MEXICO

## Abstract

Female genital mutilation (FGM) is a human rights violation that continues to affect over 86% of women and girls in Egypt. While it has declined, the practice is increasingly medicalized posing a significant challenge to abandonment efforts. This study aims to examine FGM medicalization in Egypt by exploring temporal trends, subnational patterns, underlying drivers, and the legal and policy context. We employed a mixed-methods approach, including a quantitative analysis of data from the 1995–2014 Egypt Demographic Health Surveys and the 2021 Egypt Family Health Survey, complemented by existing qualitative data among parents, healthcare providers, NGO program staff, and other stakeholders. Our findings demonstrate a marked decline in FGM prevalence over time and among younger cohorts. However, medicalization has risen sharply, reaching 83% in 2021. We identified three distinct typologies of subnational patterns in FGM prevalence and medicalization: (1) governorates with high FGM prevalence and high medicalization; (2) governorates where FGM persists but medicalization remains lower, and traditional practitioners like *dayas –* traditional cutters – are still commonly used; and (3) governorates with lower FGM prevalence but high rates of medicalization among those still undergoing the practice. Factors driving the medicalization of FGM in Egypt include persistent cultural beliefs that frame medicalized FGM as a safer option and means of beautification, parental deference to medical providers and a reliance on their “expertise” to determine the necessity for the procedure, limited awareness of FGM health consequences and legal prohibitions, and weak enforcement of penalties. To advance concrete action on medicalized FGM, a multi-level approach is essential—one that strengthens the enforcement of legal bans, integrates FGM into medical curricula and in-service training, and tailors interventions to subnational contexts based on the distinct patterns of FGM prevalence and medicalization across governorates.

## Introduction

Female genital mutilation (FGM) is a human rights violation affecting more than 200 million women and girls globally. FGM refers to all procedures involving partial or total removal of the female external genitalia or other injury to the female genital organs for non-medical reasons. The World Health Organization classifies FGM as belonging to one of four types: Type I (clitoridectomy) involves removal of all or part of the clitoris and/or the prepuce; Type II (excision) involves removal of the clitoris and the labia minora with or without removal of the labia majora; Type III (infibulation) involves removal of all of the external genitalia, and a positioning the labia to form a seal, leaving a pinhole opening for the passage of urine and blood; and Type IV, all unclassified forms, including nicking, pricking and piercing the skin covering or near the clitoris, but no removal of tissue [1].

In Egypt, FGM rates are declining, but prevalence rates remain high, with latest estimates indicating that 86% of Egyptian married women between the ages of 15 and 49 have undergone FGM [1]. Girls continue to be cut due to a confluence of reasons including entrenched beliefs about FGM’s role in protecting women’s chastity, concerns about marriageability and husbands’ preference for girls who are cut, and erroneous beliefs about FGM being a religious requirement. Increasingly, the procedure is conducted by health professionals, which has hampered abandonment efforts. Medicalization defined as “situations in which FGM is practiced by any category of health care provider, whether in a public or a private clinic, at home or elsewhere” [2] is now widespread in the country and the overwhelming majority of parents seek health providers to make the decision as to whether a girl needs to be cut [3].

The latest estimates of medicalization in Egypt indicate that 83% of FGM procedures among girls aged 0–17 are conducted by medical professionals [4]. Efforts aiming to prevent the medicalization of the practice have gained momentum. Recent legislative reforms and amendments increased penalties against health providers performing the procedure, banning them from practicing their profession and putting them at risk of imprisonment for a duration of five to 20 years depending on the severity of the harm caused. Nonetheless, to inform efforts to address the medicalization of FGM in Egypt data is urgently needed on the following: (1) reasons underlying medical professionals’ decision-making, specifically the kinds of social pressures providers face from their respective community members to perform FGM, the extent to which financial incentives matter, as well as the fundamental social norms that shape provider beliefs and attitudes towards the practice; (2) the new legal and regulatory environment, enforcement of amendments, and how the toughening of penalties against medical providers offsets social sanctions and deeply rooted social norms related to FGM.

This study aims to address some of these evidence gaps and seeks to elucidate the drivers of FGM medicalization by triangulating perspectives from community members, medical providers and non-governmental organization (NGO) staff involved in prevention and response of FGM. First, the study seeks to understand dynamics around medicalization in the context of Egypt, by analyzing most recent quantitative data on prevalence in medicalization and FGM trends over time. Secondly, the study draws on qualitative data to gather perceptions on the drivers of medicalized FGM, and efforts that aim to address the issue including recent legislative and policy reforms that criminalize the act.

## Methodology

This study utilizes a mixed methods approach that uses secondary quantitative and qualitative data to understand the state of the medicalization of FGM in Egypt – trends and prevalence, perceptions on drivers, and efforts that aim to address the medicalization of FGM in Egypt. The study was reviewed by the Johns Hopkins University School of Public Health Institutional Review Board and deemed exempt.

### Quantitative analysis

Quantitative data on the prevalence of FGM among women of reproductive age (WRA) and among survey respondents’ daughters (aged 0–17) were sourced from the publicly available 2021 Egypt’s Family Health Survey (EFHS) and a set of Egypt Demographic and Health Surveys (DHS) from 1995 to 2014 [4–9]. The former uses a comparable methodology to DHS enabling comparisons of estimates over time. The quantitative analysis examines trends on the prevalence of FGM among WRA (aged 15–49) and the incidence and medicalization of FGM among respondents’ daughters (aged 0–17). The trend in FGM prevalence over time is assessed by comparing FGM prevalence between the oldest and youngest age cohorts (aged 15–19, aged 45–49). Furthermore, the analysis explores the geographic distribution of FGM and its medicalization across governorates included in the 2021 EFHS.

### Qualitative analysis

We utilized secondary de-identified, unpublished qualitative data collected by the United Nations Population Fund (UNFPA) as part of an assessment they conducted between September 2022 and July 2023. Data was collected as part of efforts to evaluate and assess the FGM program, and understand decision-making around medicalization, the current legal government, and the community’s perspectives around FGM in three purposively selected governorates: Souhag, Minya, and Sharkia, which reflected geographic representation of upper and lower Egypt and varying FGM prevalence and degrees of medicalization. All procedures related to participant recruitment, data collection, and informed consent were implemented by UNFPA. Institutional Review Board (IRB) approval was obtained for the secondary use of these data.

Secondary qualitative data comprised of a series of (1) focus group discussions (FGDs) with parents (father and mothers of young girls); (2) in-depth interviews (IDIs) with health providers, medical syndicates, medical students, and community leaders; and (3) key informant interviews (KIIs) with stakeholders such as National Council of Women (NCW) staff, academics, and Ministry of Health and Population (MoHP) representatives and United Nations (UN) staff working on the issue of medicalization in Cairo (Table 1).

**Table 1:** Sample sizes of qualitative data sources.

Method	Participants	Sample size
Minya		
Focus Group Discussions	Fathers	15
	Mothers	16
	Doctors	6
	Nurses	6
	Medical students	11
	Other stakeholders (NCW Minya, NGO staff)	10
Souhag		
Focus Group Discussions	Fathers	8
	Mothers	17
In-depth Interviews	Doctors	3
	Nurses	3
	Medical students	3
	Other stakeholders (Community leaders, Medical syndicate representatives)	3
Sharkia		
Focus Group Discussions	Fathers	7
	Mothers	16
In-depth Interviews	Doctors	3
	Nurses	3
	Medical students	3
	Other Stakeholders (Community leaders, Medical syndicate representatives)	3
Cairo		
Key Informant Interviews	Medical syndicate representatives	4
	NCW	3
	Safe Women Clinic physicians	2
	Medical professors	3
	UNFPA consultant working on medicalization of FGM	1
	UNICEF FGM program officer	1
	MoHP representative	1
	Population Council representative	1

In the three case-study governorates, secondary data comprised a series of FGDs with parents (fathers and mothers of young girls) and IDIs with physicians, nurses, medical students, and key stakeholders (i.e., NCW staff, medical syndicate representatives, NGO workers, and community leaders) who can speak to the policy and normative issues around FGM. FGD data were available for all participant categories in Minya, but in Souhag and Sharkia, FGD data only represented parents, while interview data were used for the other participants. We also utilized data from KIIs of experts (i.e., academics, MoHP staff, UN agency representatives, and medical syndicate leadership) in Cairo to gain insights from experts in the capital about the practice of FGM and how it has evolved over time. Table 1 details participants of the three case-study governorates and at the central level in Cairo.

FGDs captured community insights about medicalization, namely about the demand for medicalized FGM, attitudes about abandonment and how these are impacted by medicalization, as well as concerns over health risks of FGM and perceptions around health risk messages. The IDIs provided the health providers’ views on motivation, drivers, knowledge, and attitudes towards medicalized FGM, along with social incentive structures that may sustain it. They also reflected medical students’ and syndicate representatives’ views on FGM in medical training, the enforcement of anti-FGM laws, and providers’ awareness of anti-FGM policies and health impacts. The insights gained from governorate-specific data collection were further triangulated with qualitative data on central staff based in Cairo to obtain policy-level perspectives on medicalized FGM, including KIIs with senior medical syndicate representatives, NCW staff, academics, MoHP representatives and UN staff working on the issue of medicalization.

## Results

### Spatial and temporal trends in the prevalence of FGM and its medicalization

First, we compare prevalence between the oldest and youngest age cohort to understand trends in the prevalence of FGM over time and identify when changes in prevalence occurred. Table 2 presents percent decrease in FGM across time in the country. The 1995, 2000, and 2005 data indicate that both age cohorts (15–19 and 45–49) were subjected to FGM at similar rates [5–7]. Only in 2008, do we start seeing a decrease in FGM rates between the two cohorts [8]. The difference is 2.6 in 2008, 7.4 in 2014, and 27.9 in 2021 [4,8,9]. The greatest change is detected in 2021 data, where prevalence of FGM differed by nearly 30% (94.4% among 45–49 vs 66.5% among 15–19) [4].

**Table 2:** Percent Decrease in FGM between Oldest (45-49 years) and Youngest (15-19 years) Cohorts of Women over the years in Egypt.

Year	Prevalence	15-19	45-49	Difference
1995	97.0	98.1	96.8	-1.3
2000	97.3	99.1	97.9	-1.2
2005	96.0	96.4	96.3	-0.1
2008	95.5	93.8	96.4	2.6
2014	92.3	87.6	95.0	7.4
2021	85.6	66.5	94.4	27.9

**Fig 1:**
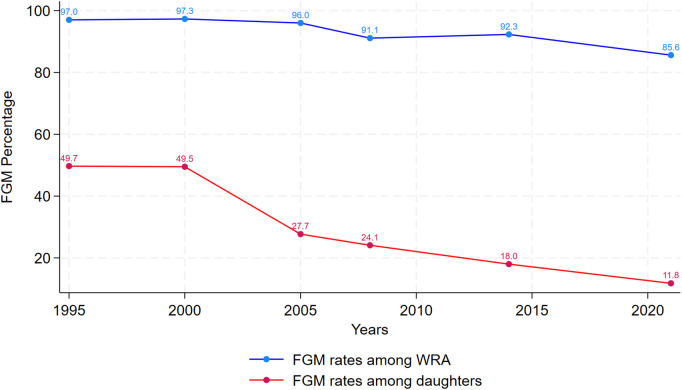
Trends in FGM prevalence among women of reproductive age (15-49) and among female respondents’ daughters in Egypt. *Note that in 1995 and 2000 the indicator used is percent of mothers with at least one daughter circumcised rather than status of all daughters, hence 1995 and 2000 FGM data among daughters are not fully comparable to subsequent years.

Fig 1 showcases trends in FGM prevalence among WRA and their daughters over time. Across time, the figure demonstrates the consistent decline in FGM prevalence among both adult female respondents and their daughters. While 97% of WRA were cut in 1995, this rate decreased to 85.6% in 2021 [4,5]. Mirroring this decline, FGM incidence among daughters decreased from 27.7% in 2005 to around 11.8% in 2021 [4,7]. It is important to note that in 1995 and 2000, the rates were calculated differently and thus the numbers are inflated for those two years. Prior to 2005, FGM rates among daughters were defined as percent of mothers with at least one daughter circumcised, whereas in subsequent years it is calculated based on the percentage of daughters aged 0–17 who are reportedly cut. Still, in the intervening years between 2005 and 2021, marked declines in FGM prevalence among daughters are still noticeable.

**Fig 2:**
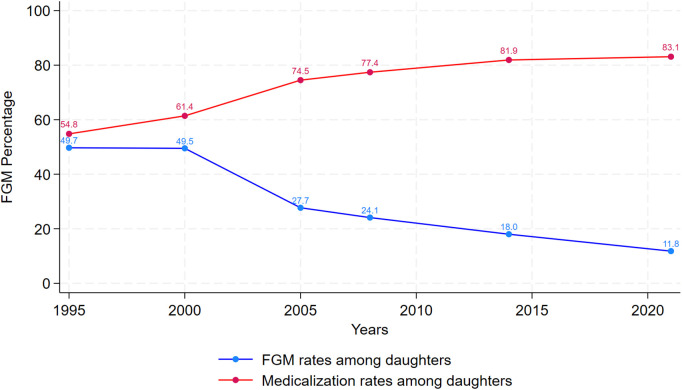
Trends in FGM rates among daughters and medicalization (also among daughters) in Egypt.

Of concern, is the rising trend in medicalization of FGM, which is demonstrated by Fig 2. While FGM incidence is decreasing substantially across time among young cohorts of girls, this has been accompanied by a consistent increase in FGM medicalization, with 2021 estimates indicating that 83% of girls are cut by medical providers compared to earlier estimates at 55% [4]. Comparing medicalization rates between survey respondents and their daughters also indicates that rates of medicalization are increasing across generations.

**Fig 3:**
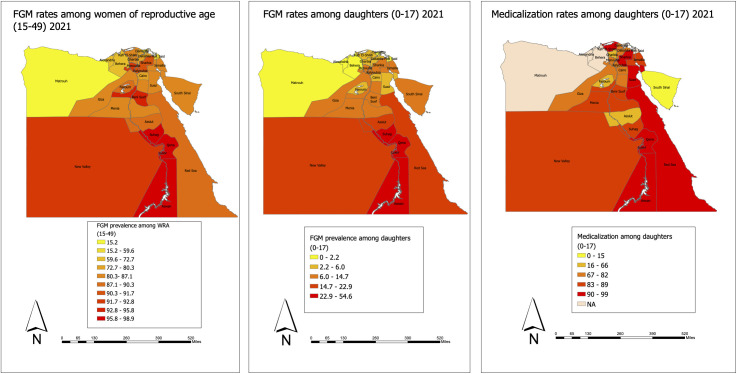
(a) Geographic distribution of FGM rates among WRA (15–4) in Egypt, 2021; (b) Geographic distribution of FGM rates among daughters (0–17) in Egypt, 2021; (c) Geographic distribution of medicalized FGM among daughters (0–17) in Egypt, 2021. Map created using ArcGIS Pro using basemap shapefile retrieved from: https://services2.arcgis.com/XSv3KNGfmrd1txPN/arcgis/rest/services/ne_10m_admin_0_countries/FeatureServer.

Recent data made available by the EFHS allows us to examine the geographic distribution of the phenomenon (Figs 3a, 3b, and 3c) [4]. FGM rates and medicalization vary substantially across governorates, with governorates in Upper Egypt having the highest rates of FGM among WRA and their daughters as well as high rates of medicalization. While lower Egypt governorates have lower FGM rates, they still have comparably high rates of medicalization (with average medicalization of 80%). Three categories of governorates emerge with respect to subnational estimates. The first group encompasses governorates with high FGM rates and high medicalization. Examples include Luxor and Aswan that have very high FGM rates as well as high rates of medicalization. In Aswan, 98% of WRA have undergone FGM and 44% of daughters have been cut. Of those, 94% have been cut by health providers. Similarly, in Luxor, FGM prevalence is 98%, more than half of daughters aged 0–17 have been cut, and 91.7% of them have been cut by health providers. Other governorates that fall into this category are Qena, Souhag, and Red Sea governorate [4].

The second group are governorates where FGM is still practiced but where medicalization is comparably lower than other governorates and *daya*s continue to be sought by communities. Those include Assiut where FGM prevalence is 89% and more than one in five daughters have already been cut. Of those, a considerable sum, 36%, are still being cut by *dayas*. Other governorates that fall into this category are Fayoum where FGM is still happening (albeit rarely) among daughters, but *dayas* are the ones predominantly doing the cutting and South Sinai where 15% of daughters are cut, but only 15% of those are cut by health professionals and the rest are cut by *dayas* [4].

The third group includes governorates with generally low FGM rates, but high medicalization. An example of this is Gharbia where prevalence has decreased to 87% and only 3.4% of daughters have been cut. However, 66% of girls who have been cut were cut by medical providers. Cairo is another governorate where prevalence has decreased substantially, but medicalization is high at 80%. Other governorates in this category are Suez, Sharkia, Dakahlia and Kalyoubia, and Kafr El-Sheikh [4].

### Drivers of medicalization in Egypt

This section presents findings from three case studies—Minya, Souhag, and Sharkia—governorates in Egypt characterized by a high prevalence of FGM medicalization. The analysis explores the micro-, meso-, and macro-level factors that contribute to the persistence of medicalization, with a focus on identifying drivers that are amenable to programmatic and policy intervention. Additionally, we present findings from interviews with key stakeholders based in the capital who bring expertise in legal and policy frameworks, medical education and regulation, and national programs addressing FGM.

#### Individual and community views around medicalization.

Interviews with parents across the three case study governorates affirmed a perceived decline in the prevalence of FGM. However, some respondents noted that the practice has become increasingly clandestine and questioned whether there was in fact a real decline or a tendency to undertake the practice in secret. While the majority of participants did not explicitly express the belief that FGM preserves girls’ honor and chastity, this rationale was still endorsed by several respondents. In Souhag, the majority of parents reported having subjected their daughters to FGM, in contrast to Minya and Sharkia, where most parents stated they had not.

Participants commonly cited health risks associated with FGM—including bleeding, infection, sexual dysfunction, reduced sexual desire, and accidental over-excision of genital tissue—as key concerns. Many viewed the involvement of health providers as a way to mitigate these risks. These views about the benefits of medicalization were most frequently expressed in Sharkia and Minya, whereas they were less commonly reported in Souhag. As noted by focus group discussion participants:

“Now parents of young girls have a lot more awareness about the complications associated with circumcising their daughters, and they are more scared for their health than before, so they just go to a medical provider rather than the daya or barber.” *–* Father

“Of course doctors are very knowledgeable and trustworthy so they will be able to act if anything goes wrong during the procedure.” *–* Father

“Doctors are more knowledgeable, experienced, and we trust them in the community.” *–* Mother

Notably, health providers were often regarded by community members as the ultimate arbiters in determining whether a girl “needed” to undergo FGM. Among parents who did not express strong personal convictions about the practice, they related relegating decision-making to medical professionals. This delegation of responsibility served as a means of conforming to prevailing social norms while at the same time distancing themselves from the moral and emotional burden of making the decision themselves.

Permeating participants accounts were the widespread perceptions of FGM as a procedure intended to “beautify” the female genitalia. Discussions frequently reflected a normative framework in which some genitalia were considered “normal” and others “abnormal.” This discourse was often linked to the perceived protrusion of the labia, with the practice of FGM being rebranded or rationalized as a form of cosmetic surgery. Such beliefs were not limited to community members but were also prevalent among medical students and physicians interviewed.

“The doctor is keen to sterilize his utilities to prevent the transmission of viruses, and use local anesthesia.”

“It is more safe with doctor, where he is able to perform the operation in a way that can reduce pain and bleeding, and he also follows up on the situation even after the operation.”

“The doctor can determine whether the girl needs to be circumcised or not, and at the same time he will not cut much of the female organ.”

“Before the doctor performs the circumcision, he asked for many lab test to know the general condition of the girl.”

“There are currently no *dayas* or others who used to practice circumcision in the past.”

According to several fathers interviewed in the study, the state’s efforts to combat FGM were undermined by the perceived inconsistency and evolution of religious edicts and advice on the issue. Participants pointed to the change in religious rulings about FGM which they said changed over time. A few participants noted that Al-Azhar, Egypt’s highest religious authority, had reduced credibility, due to what was perceived as earlier lack of opposition towards the practice. Further eroding the state’s stance, participants noted that the past allowance of FGM within health facilities contributed to public confusion and weakened arguments against medicalization.

“I don’t care what today’s *sheiks* [religious leaders] say. The *sheikhs* of the past were much better like sheikh Shaarawi who said that a girl must be cut.”

Participants were also asked about their awareness of mechanisms for reporting health providers who perform FGM. Overall, knowledge levels were low, with many individuals unaware of how or where to report such cases. A commonly held misconception centered around the belief in *talabos*—the idea that a provider must be caught in the act of performing FGM in order for legal action to be taken. This misunderstanding served as a significant disincentive to reporting, as many believed that once the act was completed, prosecution would no longer be possible. In addition to legal misconceptions, participants expressed strong concerns about the risk of being identified as the reporter. Fears of social ostracism, stigma, and retaliation—particularly in light of the close-knit communities in which participants resided —were commonly cited as deterrents to reporting

#### Health provider views around medicalization.

Health providers interviewed in the study confirmed that they are seeing fewer cases, and that requests for FGM have declined significantly. They distinguished between what they viewed as medically indicated and non-indicated FGM. Most medical students and providers interviewed said that some cases require cutting, but that it was closer to cosmetic surgery than traditional cutting. These were defined as girls whose genitalia needed “beautification” as they were “abnormal.” As put by several medical providers and students, when asked about when FGM is “warranted”:

“Some girls release excessive hormones. In those cases, their genitalia will look abnormal and the protrusion of their labia will be manifest. In those cases, the girl needs to undergo surgery.”

“Depends on how it looks, if it looks ‘harmful to the eye’ then the surgery needs to be performed.”

“In some cases, the husband does not like the way the genitalia looks. In this case, the procedure should be performed to beautify the genitalia."

“It is required if the girl has protrusions in her genitalia.”

When asked what they will do if approached by parents who want to cut their girls, providers and students commonly answered that they would first examine the girl. This response was common both among proponents of abandonment as well as those who believed in medically indicated FGM. For the first group, it was a compelling way to convince parents against performing FGM, by claiming that the girl does not need it based on the examination. For the second group, it was an important step in determining whether the genitalia were in fact “abnormal.”

Some providers interviewed espoused harmful beliefs and stated that FGM was important for ensuring girls’ chastity and cleanliness. Half of providers (doctors and nurses) across the three settings believed that FGM can be important if there is a need for it, which they defined as abnormal-looking genitalia, but few participants explicitly said that it was religiously required. For example, while almost half of doctors interviewed in Sohag agreed that FGM can be medically necessary if the genitalia is abnormal, only one explicitly said that this was a religious requirement and one of Islam’s important precepts. Among medical students interviewed in the same governorate, two medical students said the practice is a longstanding religious tradition. Among nurses in Sohag, participants were similarly divided, with around half espousing pro-FGM beliefs, citing four primary reasons for their position: (1) FGM is an inherited cultural tradition that must be upheld; (2) it is imperative for preserving girls’ chastity and protecting them from sexual wrongdoing; (3) it can be a form of beautification or a corrective procedure to improve the appearance of the genitals; and (4) it facilitates normal child delivery.

When asked about laws around FGM, there was widespread lack of awareness of the current legal framework. There was consensus among participants that legal consequences of medicalization were never addressed in medical school courses or curricula. When asked about reporting, participants overwhelmingly expressed their reluctance to report on their colleagues. If they heard that a fellow doctor working in the same clinic was performing the procedure, most participants said they would not report him/her. This was also fueled by misconceptions around *talabos*. Mirroring concerns expressed by community members, providers were discouraged from reporting on their colleagues out of fears that their identity would be disclosed, that other doctors would retaliate against them, or that they would lose the trust of their community. As expressed by one doctor “If one of my fellow doctors is performing the procedure, I would not report him. This would mean losing the trust of people in the community and of other medical doctors.”

Similarly, when asked what they would do if a parent expressed an intention to cut his/her daughter at a *daya’s*, there was a general reluctance to report the parents as well, due to fears of loss of trust in the community. Also starkly absent in providers’ responses was any reference to counseling that they would deliver to parents on the health, social, and psychological repercussions of FGM.

”It is no longer my business if they choose to go to someone else. I will not intervene.”*–* Male physician in Minya”

Many providers also lacked confidence that measures will be taken against the doctor in question. In fact, one participant recounted the case of a doctor in their community who was reported for having performed FGM on several girls but was not prosecuted because of lack of “incriminating evidence.”

#### Medical education and medical regulatory environment.

Key informants identified several challenges within the current medical education and regulatory environment that contribute to the persistence of FGM medicalization. First, they noted the absence of systematic inclusion of content on FGM and medicalization in medical curricula, as well as in general pre-service and in-service training programs. This gap, they argued, has contributed to widespread lack of awareness among health providers regarding both the harmful consequences of medicalization and the legal implications of performing FGM. While a dedicated curriculum on FGM and medicalization has reportedly been developed, at the time of data collection, participants noted that it has not been adopted consistently across all medical schools. Indeed, nearly all medical students and professors interviewed in the study confirmed that the topic is not addressed during their formal education. Informants also emphasized the broader absence of training in sexual health, including sexual pleasure, counseling, and attitude transformation—critical areas in sexual and reproductive health.

Second, in-service training for doctors was described as ad hoc and opportunistic, often driven by donor funding or program-specific initiatives rather than being part of a coordinated or sustained national strategy to reach the health workforce with anti-FGM messaging. Some key informants expressed concerns regarding current estimates of FGM medicalization. A representative from the medical syndicate stated: the participant then went on to explain:

“I have reservations about the percentage of medicalization being presented – it is high because most of those who do this say that they are doctors, and they are not doctors, especially since it cannot be proven that they are doctors. I am from Sohag governorate, which has one of the highest percentages of FGM. And when we ask them who has done it. They say a doctor. We ask them, okay, this is a doctor who has a clinic? They say, no, we don’t know. He comes in the middle of the month when the moon is full and we gather the girls for him, and he cuts them. So we hear stories like this. I am not convinced that this person is a doctor though.”

“In Souhag, we just caught a woman who was claiming to be an OBGYN who was cutting girls.”

Another informant pointed to a different concern: as medicalization increases, complications associated with FGM may become less visible, leading to fewer reports and further normalization of the practice. According to this informant, the reduction in observable harm may undermine community mobilization efforts against FGM, making abandonment increasingly difficult.

#### Legal and policy environment.

Key informants identified the recent strengthening of legal penalties against FGM as a significant advancement in efforts to combat the practice. Recent amendments to Egypt’s penal code have reclassified FGM from a misdemeanor to a felony and introduced more severe sanctions for health professionals found guilty of performing the procedure. These include prison sentences ranging from ten to fifteen years, professional disbarment for five years, and the closure of medical facilities where FGM is conducted.

According to key informants, these legal reforms have had a deterrent effect, instilling fear among some providers and raising the stakes for those who continue to engage in the practice. However, several implementation challenges persist. A key barrier is the limited awareness of anti-FGM legislation among both health professionals and the broader public. Medical providers and students across governorates demonstrated low levels of familiarity with the legal framework, and community members were similarly unaware of the specifics of the law and the consequences of violating it.

Moreover, participants consistently reported confusion and uncertainty surrounding the mechanisms for reporting FGM cases. Help lines—intended to facilitate anonymous reporting—were not widely known, even among providers. Some participants believed that filing a report required going directly to a police station, which is a time-consuming process. A particularly widespread misconception was the belief in *talabos*.

## Discussion

The present study uncovers several trends and factors driving the medicalization of FGM in Egypt, that have hindered progress towards its elimination. Population-based data show that FGM medicalization is on the rise, and while there have been substantial declines in FGM prevalence and incidence, these declines may very well be interrupted or reversed by the increasing trend in medicalization. Qualitative insights also suggest a decline in FGM demand, accompanied by a shift in the moral and decision-making responsibility toward health providers, who were increasingly viewed by community members as the key authority in determining whether a girl should undergo the practice. The literature supports this trend, highlighting a common justification for the practice framed through a harm reduction lens, arguing that it carries less risk if performed by medical professionals as opposed to traditional providers. Other common driving factors include adherence to cultural beliefs, financial incentives, and socio-communal pressures that perpetuate the practice of FGM [10]. The analysis also reveals specific patterning in FGM, and medicalization rates underscores the importance of sub-nationally differentiated approaches that reflect the complex and evolving landscape of FGM in Egypt.

Analysis of subnational data reveals three distinct typologies of governorates based on FGM prevalence and rates of medicalization, each requiring tailored intervention strategies. The first group comprises governorates with both high FGM prevalence and high medicalization—such as Luxor, Aswan, and Qena—where interventions should prioritize regulatory enforcement and targeted engagement with medical professionals, including integration of anti-FGM content in pre-service and in-service medical training. The second group includes governorates where FGM persists but medicalization remains relatively low, with traditional practitioners such as *dayas* continuing to perform the procedure. In these areas, including Assiut and Fayoum, behavior change communication (BCC) strategies should be designed to directly target traditional providers and shift community norms. The third group, exemplified by Gharbia, consists of governorates with low overall prevalence but high rates of medicalization among the small number of cases that do occur. In these settings, FGM programming must account for the changing nature of the practice, reinforcing professional accountability and ethical obligations through health sector oversight and professional associations. Moreover, our study sheds light on important sociocultural drivers of medicalized FGM. Salient in our data was the belief among participants in the need to correct perceived genitalia abnormalities. This belief, deeply rooted in cultural norms surrounding beauty and cleanliness, was a key contributing factor to the ongoing practice. Prior research has documented how Egyptian mothers consider medicalized FGM as a modern, safer alternative to traditional practice and have described selecting a doctor as a more secure, hygienic, and less harmful option, viewing the involvement of health providers as a way of protecting their daughters’ health [11]. Notably, an analysis found that the odds of utilized FGM medicalization were significantly higher among mothers with higher education levels and greater household wealth [12].

Additionally, data from the 2015 Egypt Health Issues Survey also confirm that the majority – over 80% – of both mothers and fathers believe that husbands prefer their wives circumcised, thereby reinforcing the continuation of the practice for their daughters [13]. Some families have indicated they would be satisfied with a very minimal form of cutting if their daughter is still considered circumcised, reflecting an attempt to sustain the cultural norm while ostensibly minimizing harm [14]. In our study, while health providers themselves expressed mixed views on FGM, a substantial number of doctors and nurses across all three settings (Minya, Souhag, and Sharkia) were supportive, justifying the medical indication of a “cosmetic” procedure. Similarly, a study in two hospitals in Cairo and Gharbia revealed that more than 60% of healthcare workers support the continuation of this practice and would perform it on their daughters [15]. The reframing of FGM as a “cosmetic surgery” also emerged in another study conducted in Assiut, Cairo, and Al Gharbeya [14]. Health providers reject the label of circumcision or FGM and instead refer to the practice as a form of cosmetic enhancement, otherwise known as female genital cosmetic surgery (FGCS). While in practice FGM and FGCS encompass overlapping procedures (augmentation of the clitoris, labia, or vaginal opening), they suggest differing social functions: FGC Is typically seen as a means to control women’s sexuality, while FGCS is framed as a personal choice driven by beauty ideals like minimal labia and vaginal tightening [16]. The former, however, is practiced among minors who are unable to provide informed consent and its repackaging as cosmetic surgery is a dangerous phenomenon that masks the true harm of the procedure and allows health professionals to distance themselves from the socially and legally condemned aspects of the practice [14,16,17].

Egypt’s situation mirrors a broader global pattern in countries such as Sudan, Guinea, Nigeria and Kenya where increased proportions of FGM are being carried out by formal health providers under similar pretexts of safety and cleanliness [18]. In Nigeria and Sudan, health-care professionals such as the doctors and nurses continuously carry out FGM with the aim of preventing the complications associated with traditional methods such as infections, injuries, pains, and excessive bleeding [19,20]. Furthermore, a broader global review identified medical professionals performing FGM in both public and private sectors, and even after retirement, sometimes backed by external organizations often defending medicalized FGM on the grounds of harm reduction [21]. However, medicalization is not necessarily safer, less intrusive, or less substantial, and it gives off the false impression that the procedure is safe and essentially legitimizes and normalizes the harmful practice. The justification of FGM among health providers, compounded by the reduction of reported immediate complications, is particularly concerning as it fuels the normalization of this practice. In fact, a recent systematic review and meta-analysis on the health complications of FGM, cites consistent evidence of both short- and long-term obstetric, urologic, sexual, and psychological complications over a girl’s life course [11].

Although only a small number of health providers explicitly cited religious justification for FGM, shifting and ambiguous religious rulings were found to generate uncertainty within communities and undermine anti-FGM messaging. For instance, a 2009 survey of Egyptian physicians found that over 80% did not approve of FGM, those who continued to practice it were largely swayed by financial incentives, social pressures, and perceived duty to reduce harm, while less than 15% of those that practiced FGM cited religious reasons as their motivator [22]. This ambiguity is rooted in a historical context in which Al-Azhar, Egypt’s foremost religious authority, has issued inconsistent positions on the practice. The first ministerial decree prohibiting FGM in 1996 allowed the practice to be done by health providers for medical reasons, effectively legitimizing its performance in clinical settings. While the decree was challenged in court by Islamic leaders under the pretext that it violated Sharia, the Supreme Administrative court 1997 ruling further confirmed the prohibition of FGM while retaining the exception that granted health providers final say on whether there are medical grounds for performing the procedure. This combined with the overemphasis on the health risks of FGM allowed medicalization to flourish in the country [23]. The move to ban FGM from being practiced by health professionals was not made until 2007, and the criminalization of the act only followed suit in 2008. This may be in part why we start seeing declines in FGM in the 2008 data and afterwards, with FGM rates among girls 15–19 dropping from 96.4% in 2005 to 93.8% in 2008 and further to 87.6% in 2014 and 66.5% in 2021 [4,7–9]. That said, enforcement of the decree against medicalization could be strengthened, as penalties for health care providers are often viewed as inconsistently applied or insufficiently punitive [24]. Other countries have faced similar policy challenges. For example, in Indonesia an official regulation in 2010 briefly permitted certain forms of “harmless” medicalized FGM procedures before it was rescinded due to international and domestic opposition [25]. On the other hand, Kenya has enacted robust laws that categorically forbid FGM by anyone, including healthcare professionals, however enforcement remains an ongoing struggle [18]. These examples of policy challenges in other countries highlight the need for strong legal frameworks, coupled with clarity and political will to avoid the enabling and persistence of medicalized FGM.

Another factor driving the continued practice of medicalized FGM in Egypt is the reluctance within the medical community to report colleagues who perform this practice or parents who request this procedure for their daughters. This is driven by fears of retaliation and concerns about losing the trust of the community and stems in part from misunderstandings about the anonymity of reporting. This underscores the importance of coordinated efforts and engagement among communities, health practitioners, government, and law enforcement to raise awareness around the legal and health consequences of FGM. Overall, these findings corroborate medicalization as a growing concern in Egypt and transnationally, which will continue to stall progress towards global abandonment of FGM if not comprehensively addressed.

### Recommendations for a way forward to tackle the medicalization of FGM in Egypt

At the micro-level, medicalized FGM is perceived as a safer alternative, and is being understood as a way to beautify female genitalia by mothers and fathers of young girls. Programming should thus invest in correcting misconceptions around the shape and size of female genitalia, which continue to drive the practice, and raise awareness of the dangers of FGM, including of medicalized FGM. Targeting parents of young girls and girls themselves with messaging about FGM and the fact that FGM is unsafe no matter who does it is critical in encouraging families to abandon the practice. More importantly, however, social norm programming that empowers women and girls and transforms underlying norms and conventions that operate in these contexts is crucial.

At the meso-level, community-wide discussions of medicalized FGM are needed to bring about change in community attitudes and norms around medicalized FGM. Further, to effectively end the medicalization of FGM, it is crucial to engage healthcare professionals—such as doctors, nurses, and midwives—in open discussion that emphasizes human rights, the ethical principle of “do no harm,” and their responsibility in prevention and response [26]. While integrating training of medical providers in community-based activities is crucial, pre-service and in-service training by medical professional bodies is the first step in ensuring that the health workforce advocates for FGM abandonment. In fact, research has shown glaring gaps in the training of health workers on this issue: in one Egyptian study, only 28% of medical students recognized that medicalized FGM violates professional ethics, less than 6% were able to define what FGM was, and 73% favored medicalization as a harm reduction approach [27]. This underscores the urgency of incorporating FGM, including its legal and ethical implication, into health student curriculum. Current providers should also be educated that there is no medical indication for FGM, and that examining the genitalia of young girls to determine whether FGM is needed is unethical and harmful. In Guinea, Kenya, and Somalia, implementation of the 2022 World Health Organization FGM prevention training package for primary healthcare workers, designed to strengthen their understanding of the practice and its risk, while promoting a person-centered communication approach has been linked to improved provider knowledge and a decrease in client support for, and intentions of subjecting their daughters to FGM [28,29].

Moreover, findings at the macro-level indicate that Egypt has created a conducive legislative and regulatory environment when it comes to FGM. However, more needs to be done to ensure stronger enforcement of laws and policies. This includes shifting from an overemphasis on medical consequences of the procedure, abandoning rhetoric on bleeding, infections, death, and moving towards discourse around bodily integrity and autonomy to avoid unintentionally encouraging a reliance on medical providers as a solution to medical consequences [18,30–32]. Underpinning these interventions should be a general message that FGM constitutes a human rights violation with lifelong consequences irrespective of who performs it. For one, current reporting mechanisms need to be strengthened and promoted in the community, among health providers, law enforcement agencies, and judicial bodies. It is important to concurrently strengthen the anonymity of reporting to protect those who report from retaliation from peers and community members. Penalizing parents for intention to cut or for cutting their daughters however is one issue that merits careful consideration as it has been associated with unintended consequences. The current legal framework not only penalizes medical providers but also parents, which participants said deters reporting of providers because it may implicate parents as well. There is some evidence that suggests that penalizing parents (not just in relation to the crime of FGM but in relation to other crimes such as child marriage) does little to deter the practice and may even be counterproductive as it disincentivizes reporting, encourages secrecy and strategies to evade detection by authorities, and separates families [33,34].

In addition to strengthening legislation and enforcement of bans on medicalized FGM, the country currently has a national strategy that includes structured multisectoral activities on FGM and medicalization, which is an important pre-requisite to galvanizing political commitment towards abandonment. However, without financial investment and planning, the strategy cannot be implemented. Domestic financial investment in FGM prevention is thus needed to concretize action on FGM.

### Limitations

This study should be considered in light of limitations. First, the qualitative component of the study relied on secondary qualitative data from three governorates selected purposively according to preset criteria. While this approach yielded rich and in-depth insights, the extent to which findings can be generalized to other governorates is uncertain. However, findings may very well apply to other governorates in Upper and Lower Egypt with high FGM medicalization rates where similar social norms and conventions operate. Additionally, some of the results are relevant nationally, because they concern the overarching legal framework and supportive environment. Secondly, we were constrained by the available quantitative data on FGM prevalence and medicalization rates. There was notable heterogeneity in the methodologies used by the DHS surveys which reduced from comparability of the estimates available. Additionally, the authors did not have access to the full EFHS raw data, and the full report was not yet drafted at the time this report was written which precluded calculation of estimates needed to draw comparisons with existing data sources. Finally, the scope of the study was limited to medicalization and so the study was ill-suited to answer more general questions about FGM generally and how best to prevent it.

## Conclusion

The study highlights the rise in medicalization amid the steady overall decline in FGM in Egypt. The current legislative environment supports the elimination of FGM, criminalizing the practice even when performed by healthcare providers. However, persistent cultural beliefs that frame medicalized FGM as a safer alternative and a way to beautify the female genitalia, along with a heavy reliance on healthcare providers to determine its necessity, must be critically addressed.
